# Blood pressure trajectory of inpatient stroke rehabilitation patients from the Determining Optimal Post-Stroke Exercise (DOSE) trial over the first 12 months post-stroke

**DOI:** 10.3389/fneur.2023.1245881

**Published:** 2023-09-19

**Authors:** Stanley H. Hung, Christopher Tierney, Tara D. Klassen, Amy Schneeberg, Mark T. Bayley, Sean P. Dukelow, Michael D. Hill, Andrei Krassioukov, Sepideh Pooyania, Marc J. Poulin, Jennifer Yao, Janice J. Eng

**Affiliations:** ^1^Department of Physical Therapy, University of British Columbia, Vancouver, BC, Canada; ^2^Rehabilitation Research Program, Center for Aging SMART, Vancouver Coastal Health Research Institute, Vancouver, BC, Canada; ^3^painPRO Clinics, Vancouver, BC, Canada; ^4^Division of Physical Medicine and Rehabilitation, Department of Medicine, University of Toronto, Toronto, ON, Canada; ^5^Department of Clinical Neurosciences and Hotchkiss Brain Institute, University of Calgary, Calgary, AB, Canada; ^6^Division of Physical Medicine and Rehabilitation, Department of Medicine, University of British Columbia, Vancouver, BC, Canada; ^7^Division of Physical Medicine and Rehabilitation, University of Manitoba, Winnipeg, MB, Canada; ^8^Department of Physiology and Pharmacology and Hotchkiss Brain Institute, University of Calgary, Calgary, AB, Canada

**Keywords:** stroke, rehabilitation, blood pressure, hypertension, exercise, recovery

## Abstract

**Background:**

High blood pressure (BP) is the primary risk factor for recurrent strokes. Despite established clinical guidelines, some stroke survivors exhibit uncontrolled BP over the first 12 months post-stroke. Furthermore, research on BP trajectories in stroke survivors admitted to inpatient rehabilitation hospitals is limited. Exercise is recommended to reduce BP after stroke. However, the effect of high repetition gait training at aerobic intensities (>40% heart rate reserve; HRR) during inpatient rehabilitation on BP is unclear. We aimed to determine the effect of an aerobic gait training intervention on BP trajectory over the first 12 months post-stroke.

**Methods:**

This is a secondary analysis of the Determining Optimal Post-Stroke Exercise (DOSE) trial. Participants with stroke admitted to inpatient rehabilitation hospitals were recruited and randomized to usual care (*n* = 24), DOSE1 (*n* = 25; >2,000 steps, 40–60% HRR for >30 min/session, 20 sessions over 4 weeks), or DOSE2 (*n* = 25; additional DOSE1 session/day) groups. Resting BP [systolic (SBP) and diastolic (DBP)] was measured at baseline (inpatient rehabilitation admission), post-intervention (near inpatient discharge), 6- and 12-month post-stroke. Linear mixed-effects models were used to examine the effects of group and time (weeks post-stroke) on SBP, DBP and hypertension (≥140/90 mmHg; ≥130/80 mmHg, if diabetic), controlling for age, stroke type, and baseline history of hypertension.

**Results:**

No effect of intervention group on SBP, DBP, or hypertension was observed. BP increased from baseline to 12-month post-stroke for SBP (from [mean ± standard deviation] 121.8 ± 15.0 to 131.8 ± 17.8 mmHg) and for DBP (74.4 ± 9.8 to 78.5 ± 10.1 mmHg). The proportion of hypertensive participants increased from 20.8% (*n* = 15/72) to 32.8% (*n* = 19/58). These increases in BP were statistically significant: an effect [estimation (95%CI), value of *p*] of time was observed on SBP [0.19 (0.12–0.26) mmHg/week, *p* < 0.001], DBP [0.09 (0.05–0.14) mmHg/week, *p* < 0.001], and hypertension [OR (95%CI): 1.03 (1.01–1.05), *p* = 0.010]. A baseline history of hypertension was associated with higher SBP by 13.45 (8.73–18.17) mmHg, higher DBP by 5.57 (2.02–9.12) mmHg, and 42.22 (6.60–270.08) times the odds of being hypertensive at each timepoint, compared to those without.

**Conclusion:**

Blood pressure increased after inpatient rehabilitation over the first 12 months post-stroke, especially among those with a history of hypertension. The 4-week aerobic gait training intervention did not influence this trajectory.

## 1. Introduction

Recurrent strokes account for 25–30% of all strokes and represent unsuccessful secondary prevention (1). Stroke survivors are at high risk for subsequent adverse cardiovascular events, including stroke, myocardial infarction, and vascular death, with the largest risk occurring within the first 12 months post-stroke (up to 11.1%) (2, 3). Preventing these subsequent adverse cardiovascular events is a key priority in post-stroke care (4). High blood pressure (BP) is the single most important modifiable risk factor for primary and secondary stroke prevention (4). Accordingly, there is strong evidence from meta-analyses of randomized controlled trials that lowering BP in stroke survivors reduces the risk of recurrent stroke (5–7). Clinical guidelines have been developed to help manage and control BP after stroke (4, 8). In general, healthcare teams are recommended to frequently review (e.g., monthly) BP early after stroke until targets and optimal therapies are achieved (4). Furthermore, quantitative modeling suggests that effective, early secondary prevention strategies, including adherence to three types of medications (aspirin, a statin, and an antihypertensive medication), exercise and dietary changes, could prevent 80% of recurrent cardiovascular events after stroke (9). Despite these established recommendations and guidelines, controlling BP after stroke remains challenging with studies reporting that many stroke survivors continue to have elevated and uncontrolled BP over the first 12 months post-stroke (10–15). Furthermore, these studies primarily recruited people from the acute hospitalization setting. Therefore, there is limited research describing BP trajectories in an inpatient stroke rehabilitation population over the first 12 months post-stroke. Patients who receive inpatient stroke rehabilitation services represent patients with moderate to severe impairments in physical and/or cognitive function and may be at higher risk of facing challenges with managing secondary prevention (e.g., participating in physical activity or managing medications). Understanding the BP trajectory of stroke survivors from an inpatient rehabilitation setting may help inform BP management strategies specifically for stroke survivors requiring rehabilitation.

Exercise is a recommended strategy to reduce BP after stroke for secondary prevention (16). Wang and colleagues conducted a meta-analysis (20 randomized controlled trials, *n* = 1,031) and reported that aerobic exercise interventions can reduce BP in transient ischaemic attack (TIA) and stroke survivors (17). In particular, interventions that begin within 6 months after stroke or TIA produced the greatest reductions in BP (17). Furthermore, aerobic exercise at an intensity >40% heart rate reserve (HRR) is recommended to reduce BP, and evidence suggests that higher intensity exercise (e.g., >60% HRR) may provide greater reductions in BP (18). Therefore, inpatient stroke rehabilitation may be an opportune setting to provide aerobic exercise interventions to reduce BP after stroke. There is growing evidence that stroke rehabilitation interventions that involve high repetition gait training at aerobic intensities (e.g., >40% HRR) are superior to conventional forms of therapy in improving walking speed, walking endurance, balance, and quality of life after stroke (19–22). However, the effect of these high repetition gait training interventions at aerobic intensities on BP remain unclear. The Determining Optimal Post-Stroke Exercise (DOSE) randomized controlled trial showed evidence of greater improvements in walking recovery among those receiving high repetition gait training at aerobic intensities (i.e., progression to at least 2,000 steps and at least 30 min of 40–60% HRR per session) during inpatient rehabilitation, compared with usual care (20, 23). In this secondary analysis, we aimed to determine the effect of high repetition gait training at aerobic intensities on the BP trajectory over the first 12 months post-stroke in participants from the DOSE trial.

## 2. Methods

The protocol, procedures, and main results of the DOSE trial have been previously described (20, 24). In brief, the DOSE trial was a multi-site, phase II, assessor-blinded, randomized controlled trial recruiting participants between 2014 and 2018 from six inpatient rehabilitation hospitals across Canada. Ethical approvals were obtained from the university and hospital institutional review boards of each respective study site. Participants were required to provide written informed consent.

### 2.1. Patient population

Between March 2014 and July 2018, 2,387 patients were admitted to the participating study sites with stroke. Of which, 2,141 were consecutively screened and assessed for study eligibility. Inclusion criteria were: adults who were within 10 weeks post-stroke with lower extremity hemiparesis (<4/5 manual muscle grade in at least one of the major lower extremity muscles using the Medical Research Council scale), pre-stroke disability <2 on the Modified Rankin Scale, ability to ambulate ≥5 meters with up to one person maximum assist and assistive/orthotic device as required, over-ground walking speed of <1.0 m/s, able to follow directions, and successful completion of a graded exercise stress test using criteria set out by the American College of Sports Medicine (25). Excluded participants had a pre-stroke health condition including a serious medical or painful condition (e.g., active cancer), another neurological condition, a gait disorder, or had enrolled in a drug or another exercise rehabilitation program.

### 2.2. Usual care and intervention groups

Participants were randomized to one of three groups (Usual Care, DOSE1, or DOSE2) on a 1:1:1 ratio, stratified by age (<60 or ≥ 60), using a fully concealed internet-based dynamic allocation randomization that was generated in real-time. The Usual Care group received standard inpatient physical therapy which progressed upper and lower limb functional exercises as tolerated and typically provided over 5, 1-h sessions per week, until the participant was discharged (normally after 4–6 weeks of inpatient rehabilitation). The DOSE1 group received an intervention that replaced standard inpatient physical therapy session for a total of 20 sessions (1 h/day, 5 days/week, for 4 weeks). The therapist progressed the participants to complete a minimum of 30 min at an intensity ≥40% HRR, gradually progressing to >60% HRR, and achieve >2000 walking steps per session by the end of the 4-week intervention. The DOSE2 group received an intervention that consisted of DOSE1 activities (typically in the morning) and received a similar second session later in the day (typically from 4 to 5 pm daily).

Detailed description of the intervention fidelity has been previously reported (20). In brief, over the 4-week intervention period, attendance to the planned therapy sessions were 226/240 (94%) sessions for the Usual Care group, 494/500 (99%) sessions for the DOSE1 group, and 904/960 (94%) sessions for the DOSE2 group. On average (mean ± SD), the total minutes spent in aerobic intensities (≥40% HRR) for each therapy session were 11 ± 9 min in the Usual Care group, 27 ± 11 min in the DOSE1 group, and 52 ± 24 min in the DOSE2 group.

### 2.3. Outcomes

Study outcomes were assessed at four timepoints: baseline at rehabilitation admission (2–10 weeks post-stroke), post-intervention (after the 4-week intervention), and 6- and 12-month post-stroke. Participant characteristics were collected at the baseline evaluation, including age, sex, date and type of stroke, and self-reported history of hypertension or diabetes (i.e., medical diagnosis or use of relevant medication any time prior to study enrolment). Stroke severity [National Institutes of Health Stroke Scale (26); NIH Stroke Scale] was assessed by a blinded-assessor at the baseline evaluation.

Blood pressure was assessed and recorded as a screening tool for the primary outcome measure of the 6 min walk test (6MWT) at each timepoint. Systolic BP (SBP) and diastolic BP (DBP) were assessed in concordance with the body positions recommended by the Hypertension Canada Guidelines (27). Participants were first seated at rest in a chair for 5–10 min prior to the evaluation commencing. The Montreal Cognitive Assessment was then evaluated, which took approximately 10 min. BP was then taken by a trained, blinded assessor using an electronic (oscillometer) upper arm device standard to the inpatient rehabilitation unit or outpatient clinic in which the evaluation took place. BP was measured with the participant in a sitting position with their arm supported at the level of the heart. Only one BP measurement was taken. The presence of hypertension at each timepoint was defined as SBP ≥140 mmHg or DBP ≥90 mmHg, except for patients with a history of diabetes, where the presence of hypertension was defined as SBP ≥130 mmHg or DBP ≥80 mmHg (27).

### 2.4. Data analysis

Statistical analyses were conducted using the R computing environment (version 4.2.2., R Core Team, 2022), with an alpha of 0.05. Three linear mixed-effects models were undertaken to first determine whether there was an effect of group (Control, DOSE1, and DOSE2) and the four timepoints on (1) SBP and (2) DBP and on (3) presence of hypertension. Subsequently, longitudinal modeling was undertaken for SBP using linear mixed-effects modeling, with a random effect for each participant. The null hypothesis tested that the SBP remained the same over time. Time was included as a continuous variable to allow for a non-linear slope of recovery. Time since stroke (weeks), age (centered to the mean; years), stroke type (ischemic or hemorrhagic), and baseline history of hypertension (yes/no) were included in all models. A separate linear mixed-effects model was undertaken to estimate potential interaction effects between time since stroke and history of hypertension on SBP, including age (years) and stroke type (ischemic or hemorrhagic). These longitudinal models were repeated with DBP and the presence/absence of hypertension. The package lme4: Linear Mixed-Effects Models used “Eigen” and S4 (R software), which utilizes all available data and has been demonstrated to be valid in the presence of data missing at random (28).

A sensitivity analysis to investigate the impact of influential observations was conducted. All observations with a Cook’s D value equal to or greater than four times the mean were excluded, and the models were rerun. To explore potential mechanisms of missing data at 6- and 12-month post-stroke, bivariable logistic regression was used to examine the associations between demographic (age, sex) and baseline SBP to those who completed and did not complete follow-up assessment at 6- and 12-month post-stroke (yes/no).

## 3. Results

**Figure 1:**
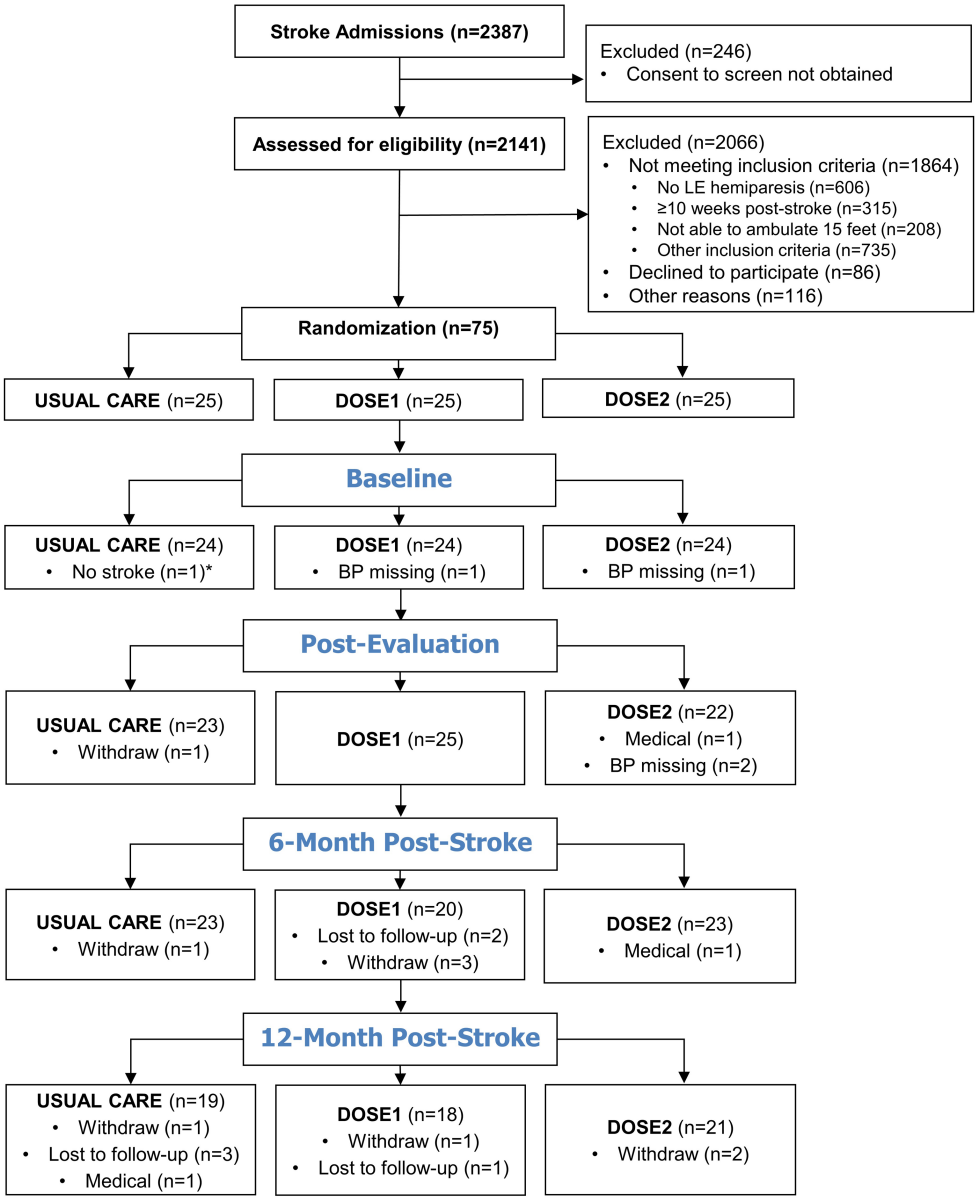
Participant recruitment and blood pressure data availability flow diagram. *After study completion, one Usual Care participant was found to not have a primary stroke diagnosis and did not meet the inclusion criteria. Therefore, this participant’s data was not included in the demographic or statistical analyses.

Figure 1 illustrates the recruitment and availability of BP data at each time point. The most common reason for study exclusion was a lack of lower extremity hemiparesis from over one-quarter screened. Among all participants and timepoints, in addition to missing BP data from study withdrawal (*n* = 9), study drop-out for medical reasons (*n* = 3), and loss to follow-up (*n* = 6), BP measurements were missed at the attended study visits on four occasions. Seventy-four participants were included in the current study, and their characteristics are summarized in Table 1. The overall sample had moderate-to-severe physical deficits at baseline with a mean 6MWT distance of 132.0 m (i.e., average 0.36 m/s gait speed). Of note, a median NIH Stroke Scale of 4.0 typically represents a mild stroke when assessed immediately after an acute stroke, but this assessment was done, on average, 4 weeks post-stroke in individuals requiring rehabilitation. Among all participants, from baseline to 12-month post-stroke, the mean ± SD of SBP increased from 121.8 ± 15.0 to 131.8 ± 17.8 mmHg and the DBP from 74.4 ± 9.8 to 78.5 ± 10.1 mmHg. The proportion of hypertensive participants increased from 20.8% (*n* = 15/72) to 32.8% (*n* = 19/58).

**Table 1:** Participant characteristics and blood pressure data.

Characteristics	All groups (*n* = 74)	Usual Care (*n* = 24)	DOSE1 (*n* = 25)	DOSE2 (*n* = 25)
Age (years), mean ± SD	57.0 ± 11.4	57.6 ± 13.0	56.0 ± 11.4	57.5 ± 10.0
Male sex, *n* (%)	44 (59.5)	14 (58.3)	16 (64.0)	14 (56.0)
History of hypertension^¥^, *n* (%)	49 (66.2)	13 (54.2)	15 (60.0)	21 (84.0)
History of diabetes^¥^, *n* (%)	20 (27.0)	6 (25.0)	8 (32.0)	6 (25.0)
Time from stroke to randomization (days), mean ± SD	27.1 ± 10.4	25.8 ± 11.0	26.9 ± 10.3	28.6 ± 10.3
Side of hemiparesis, *n* (%)	L = 42; R = 32	L = 16; R = 8	L = 10; R = 15	L = 16; R = 9
Type of stroke, *n*	Ischemic = 61 Hemorrhagic = 13	Ischemic = 20 Hemorrhagic = 4	Ischemic = 22 Hemorrhagic = 3	Ischemic = 19 Hemorrhagic = 6
Stroke location, *n*	Cortical = 15 Sub-Cortical = 58 Missing Data = 1	Cortical = 5 Sub-Cortical = 18 Missing Data = 1	Cortical = 4 Sub-Cortical = 21 Missing Data = 0	Cortical = 6 Sub-Cortical = 19 Missing Data = 0
NIH stroke scale (at rehabilitation baseline), median (Q1–Q3)	4.0 (3.0–7.0)	4.5 (3.0–6.5)	4.0 (3.0–7.0)	4.0 (4.0–6.0)
6MWT distance (m), mean ± SD	132.0 ± 89.6	129.2 ± 77.6	128.8 ± 97.3	137.9 ± 95.5
Blood pressure measurements				
Presence of hypertension, *n* (%)^*^				
Baseline	15/72 (20.8)	4/24 (16.7)	7/24 (29.2)	4/24 (16.7)
Post-intervention	17/70 (24.3)	5/23 (21.7)	7/25 (28.0)	5/22 (22.7)
6-month post-stroke	22/66 (33.3)	9/23 (39.1)	6/20 (30.0)	7/23 (30.4)
12-month post-stroke	19/58 (32.8)	5/19 (26.3)	6/18 (33.0)	8/21 (38.1)
Systolic blood pressure, mmHg, mean ± SD				
Baseline	121.8 ± 15.0	122.1 ± 15.3	123.4 ± 17.5	119.8 ± 11.2
Post-intervention	126.0 ± 11.5	125.8 ± 14.3	125.3 ± 10.4	127.0 ± 9.9
6-month post-stroke	128.4 ± 13.8	128.5 ± 13.7	126.4 ± 12.4	130.2 ± 15.4
12-month post-stroke	131.8 ± 17.8	132.4 ± 20.0	131.6 ± 18.4	130.7 ± 16.0
Diastolic blood pressure, mmHg, mean ± SD				
Baseline	74.4 ± 9.8	76.4 ± 11.8	74.3 ± 9.1	72.5 ± 8.0
Post-intervention	74.7 ± 9.0	73.1 ± 9.5	73.6 ± 9.1	77.7 ± 7.9
6-month post-stroke	77.3 ± 9.4	77.9 ± 9.6	76.3 ± 9.5	77.7 ± 9.5
12-month post-stroke	78.5 ± 10.1	78.6 ± 10.8	76.1 ± 9.2	80.7 ± 10.4

^¥^Prior history of hypertension or diabetes was confirmed based on self-report and current relevant medications; ^*^n is expressed as a fraction of available data. 6MWT, 6-min walk test; NIH, National Institutes of Health; Q1, 1st Quartile; Q3, 3rd Quartile.

Table 2 describes the effect of intervention group on BP over the first 12 months post-stroke. No effect of intervention group on SBP, DBP, or presence of hypertension was observed. There was, however, an effect [estimated effect (95% CI)] of time on SBP, DBP, and presence of hypertension. Compared to baseline, the post-intervention SBP increased by 4.56 (0.83–8.28) mmHg, the 6-month post-stroke SBP by 7.09 (3.30–10.88) mmHg, and the 12-month post-stroke by 10.86 (6.92–14.81) mmHg. By 12 months post-stroke, there was almost a 4-fold increase in risk of developing hypertension compared to baseline (odds ratio: 3.86, 95% CI: 1.28–11.66).

**Table 2:** Effect of intervention group on blood pressure over the first 12 months post-stroke.

Predictors	Systolic blood pressure			Diastolic blood pressure			Hypertension		
	Est.	95% CI	*p*	Est.	95% CI	*p*	Odds ratio	95% CI	*p*
Intercept	121.86	116.84–126.88	**<0.001**	74.76	71.46–78.05	**<0.001**	0.07	0.02–0.34	**0.001**
Group (DOSE1)^*^	−0.16	−6.51 to 6.20	0.961	−1.40	−5.58 to 2.77	0.508	1.55	0.28–8.54	0.612
Group (DOSE2)^*^	−0.38	−6.71 to 5.95	0.906	0.29	−3.86 to 4.45	0.890	0.90	0.16–5.01	0.900
Post-intervention^¥^	4.56	0.83–8.28	**0.017**	0.36	−2.07 to 2.79	0.770	1.47	0.52–4.16	0.467
6-month stroke^¥^	7.09	3.30–10.88	**<0.001**	3.08	0.60–5.55	**0.015**	3.41	1.19–9.79	**0.023**
12-month post-stroke^¥^	10.86	6.92–14.81	**<0.001**	4.45	1.88–7.03	**0.001**	3.86	1.28–11.66	**0.017**
Random effects									
*σ* ^2^	125.31			53.42			3.29		
τ_00 ID_	90.94			39.37			5.73		
ICC	0.42			0.42			0.64		
*N*_ID_	74			74			74		
Observations	266			266			266		
Marginal *R*^2^/Conditional *R*^2^	0.066/0.459			0.042/0.448			0.039/0.649		

^*^Reference is the usual care group. ^¥^Reference is baseline timepoint. Bold values indicate statistical significance at *p* < 0.05.

Given that no effect of intervention group on BP was observed, all participants were pooled in the longitudinal analysis. Table 3 summarizes the results from the longitudinal analysis of BP changes using linear mixed-effects models over the first 12 months post-stroke. Of note, there was an increase in SBP of 0.19 (0.12–0.26) mmHg per week post-stroke and an increase in DBP of 0.09 (0.05–0.14) mmHg per week post-stroke. There was an estimated increase in the odds of hypertension by 3% for each week post-stroke. Being older at baseline had a significant effect on DBP only, where there was a 0.21 (0.06–0.36) mmHg decrease in DBP for each 1-year of older age. Participants with a baseline history of hypertension (*n* = 49, 66.2%) were associated with higher SBP by 13.45 (8.73–18.17) mmHg, higher DBP by 5.57 (2.02–9.12) mmHg, and 42.22 (6.60–270.08) times the odds of being hypertensive at each timepoint, compared to those without. An interaction effect was observed between time after stroke and history of hypertension on DBP, where participants with history of hypertension were associated with an increase in 0.10 (0.00–0.19) mmHg per week post-stroke, and not for those without a history of hypertension (Table 4). However, no significant interaction effects were observed between time after stroke and history of hypertension for SBP and presence of hypertension. Table 5 describes the BP trajectory of participants with and without a baseline history of hypertension. Of note, participants with a history of hypertension at baseline increased their SBP from 126.4 ± 14.2 to 138.0 ± 18.5 mmHg from baseline to 12-month post-stroke, and DBP from 75.0 ± 10.4 to 81.7 ± 10.2 mmHg. Participants without a history of hypertension increased their SBP from 113.2 ± 12.6 to 120.8 ± 9.2 mmHg, while their DBP remained stable from 73.1 ± 8.5 to 72.7 ± 7.0 mmHg. Finally, the proportion of participants with a history of hypertension who were measured with the presence of hypertension increased from 13/47 (27.6%) to 19/37 (51.4%), while the proportion of those without a history of hypertension were low and remained stable.

**Table 3:** Longitudinal analyses of blood pressure from the linear mixed effects model over the first 12 months post-stroke.

Predictors	Systolic blood pressure			Diastolic blood pressure			Hypertension		
	Est.	95% CI	*p*	Est.	95% CI	*p*	Odds ratio	95% CI	*p*
(Intercept)	114.69	110.67–118.71	**<0.001**	70.61	67.65–73.57	**<0.001**	0.01	0.00–0.06	**<0.001**
Time in weeks since stroke	0.19	0.12–0.26	**<0.001**	0.09	0.05–0.14	**<0.001**	1.03	1.01–1.05	**0.010**
History of hypertension^*^	13.45	8.73–18.17	**<0.001**	5.57	2.02–9.12	**0.002**	42.22	6.60–270.08	**<0.001**
Age in years	0.06	−0.14 to 0.25	0.576	−0.21	−0.36 to −0.06	**0.006**	1.00	0.94–1.06	0.946
Hemorrhagic stroke^¥^	−4.01	−9.68 to 1.66	0.165	−1.15	−5.40 to 3.10	0.595	0.29	0.06–1.39	0.120
Random effects									
σ^2^	127.32			53.16			3.29		
τ_00 ID_	44.42			31.34			2.99		
ICC	0.27			0.37			0.48		
*N*_ID_	74			74			74		
Observations	266			266			266		
Marginal *R*^2^/Conditional *R*^2^	0.241/0.444			0.123/0.449			0.345/0.657		

^*^Reference is no history of hypertension; ^¥^Reference is ischemic stroke. Bold values indicate statistical significance at *p* < 0.05.

**Table 4:** Longitudinal analyses of blood pressure from the linear mixed effects model, including time by history of hypertension interaction, over the first 12 months post-stroke.

Predictors	Systolic blood pressure			Diastolic blood pressure			Hypertension		
	Est.	95% CI	*p*	Est.	95% CI	*p*	Odds ratio	95% CI	*p*
(Intercept)	115.98	111.42–120.55	**<0.001**	72.01	68.74–75.27	**<0.001**	0.03	0.00–0.22	**0.001**
Time in weeks since stroke	0.13	0.01–0.25	**0.032**	0.03	−0.05 to 0.11	0.413	0.97	0.91–1.04	0.401
History of hypertension^*^	11.48	5.71–17.25	**<0.001**	3.44	−0.69 to 7.57	0.102	11.12	1.34–92.59	**0.026**
Time in weeks since stroke × History of hypertension	0.09	−0.06 to 0.24	0.242	0.10	0.00–0.19	**0.049**	1.06	0.99–1.14	0.082
Age in years	0.06	−0.14 to 0.25	0.574	−0.21	−0.36 to −0.06	**0.006**	1.00	0.94–1.06	0.970
Hemorrhagic Stroke^¥^	−4.05	−9.72 to 1.62	0.161	−1.20	−5.45 to 3.05	0.579	0.28	0.06–1.38	0.117
Random effects									
*σ* ^2^	127.27			52.40			3.29		
τ_00 ID_	47.23			31.48			3.37		
ICC	0.27			0.38			0.51		
*N*_ID_	74			74			74		
Observations	266			266			266		
Marginal *R*^2^/Conditional *R*^2^	0.236/0.443			0.132/0.458			0.336/0.672		

^*^Reference is no history of hypertension; ^¥^Reference is ischemic stroke. Bold values indicate statistical significance at *p* < 0.05.

**Table 5:** Blood pressure of participants with and without a baseline history of hypertension.

Study timepoint	With history of hypertension (*n* = 49)	Without history of hypertension (*n* = 25)
Hypertension, *n* (%)^*^		
Baseline	13/49 (27.5)	2/25 (8.0)
Post-intervention	16/46 (34.8)	1/24 (4.2)
6-month post-stroke	20/42 (47.6)	2/24 (8.3)
12-month post-stroke	19/37 (51.4)	0/21 (0.0)
Systolic blood pressure, mmHg, mean ± SD		
Baseline	126.4 ± 14.2	113.2 ± 12.6
Post-intervention	129.9 ± 10.6	118.5 ± 9.3
6-month post-stroke	132.4 ± 14.3	121.5 ± 9.7
12-month post-stroke	138.0 ± 18.5	120.8 ± 9.2
Diastolic blood pressure, mmHg, mean ± SD		
Baseline	75.0 ± 10.4	73.2 ± 8.5
Post-intervention	76.1 ± 9.8	72.0 ± 7.8
6-month post-stroke	77.7 ± 9.3	76.8 ± 9.8
12-month post-stroke	81.7 ± 10.2	72.8 ± 7.0

^*^n is expressed as a fraction of available data.

A sensitivity analysis was undertaken to investigate the impact of influential observations, where 13, 11, and 3 observations with a Cooks D value equal to or greater than four times the mean were excluded for SBP, DBP, and presence of hypertension, respectively. In the re-analyses, no clinically meaningful differences in magnitudes of coefficients in the models with and without influential observations were observed (Supplementary Tables 1, 2). For missing data, 16 participants did not complete 6- and 12-month follow-up assessments. Participant age, sex, and baseline SBP were not associated with loss to follow-up (Supplementary Table 3).

## 4. Discussion

Among patients undergoing inpatient stroke rehabilitation, we did not observe an effect of the aerobic gait training interventions on BP. This is contrary to meta-analyses demonstrating an effect of exercise interventions (primarily cycling and treadmill exercise interventions) on reducing SBP (29), with potentially greater effect from interventions commencing within 6 months post-stroke (17). However, these were not conducted during inpatient rehabilitation. Providing the intervention within an inpatient rehabilitation setting may explain why no effect of the aerobic interventions was observed. Clinicians in the inpatient unit typically monitor BP daily, adjust BP medications accordingly if the BP is persistently out of range, and ensure excellent adherence to medications for these patients. The effect of this closely monitored and controlled BP may have masked the effect of the DOSE interventions, and potentially explain why we did not observe significant between-group differences in BP. Another reason may be that the exercise prescription of the DOSE intervention was not adequate to reduce SBP. The intensity (≥40% HRR), session length (60-min sessions with at least 30 min in aerobic intensities), and frequency (DOSE1: five sessions per week; DOSE2: 10 sessions per week) of the DOSE interventions were similar to the prescriptions of aerobic exercise interventions in previous randomized controlled trials that resulted in SBP reductions after stroke or TIA (17, 29). However, all interventions that started within 6 months post-stroke all reported exercise intensities ≥50% HRR and up to 85% HRR (17). Therefore, the intensity of the DOSE interventions may not have been adequate to reduce BP. Furthermore, the 4-week duration of the DOSE intervention was substantially shorter than previous randomized controlled trials (8–24 weeks), but these trials were not conducted in inpatient settings (17, 29). The prescription of the DOSE intervention was designed to improve walking recovery after stroke with a strong consideration to feasibly implement within usual care inpatient rehabilitation settings and timeframes (30). According to the Canadian Institutes for Health Information, between 2021 and 2022, the length of stay for inpatient stroke rehabilitation patients was 4 weeks (median 28.0 days), and has been gradually decreasing since 2015 (median 30.0 days) (31). Therefore, an intervention duration of at least 8 weeks within only an inpatient rehabilitation setting may not be possible. Given this, continuing exercise [e.g., community-based Fitness and Mobility Exercise (FAME) (32) or Together in Movement and Exercise (TIME™) (33) programs] or physical activity [e.g., self-managed telehealth Stroke Coach programs (34)] interventions after discharge may be the most feasible scenario to fulfill the desired intervention durations beyond the inpatient rehabilitation phase. Previous trials that also included a health education component demonstrated greater reductions in BP (17). Although patients receive incidental health education as part of usual care during inpatient rehabilitation, formal health education was not a part of the DOSE intervention (24). Further investigation would be needed to explore the role of these combined hospital and community interventions, including inpatient rehabilitation and high repetition gait training at aerobic intensities, on BP after stroke.

The current study presents a unique dataset that describes BP trajectories in a clinically distinct group of stroke survivors discharged from acute hospitalization with moderate-to-severe physical deficits requiring rehabilitation (~20% of stroke survivors admitted for acute hospitalization) (35). All studies to-date describing BP trajectories over the 12 months after stroke recruited individuals from acute hospitalization for stroke (10–14), where the majority of people are discharged to home or long term care facilities (~77%) (35). In the current study, the average BP and proportion of participants with hypertension were the lowest while participants were still living in the inpatient rehabilitation unit (i.e., at baseline and post-intervention timepoints). This is likely due to the frequent BP monitoring and high adherence to BP medications during inpatient rehabilitation. After the participants were discharged home, the proportion of participants with hypertension increased from 21% at baseline to over 30% at the 6- and 12-month post-stroke timepoints. This overall increase in hypertensive participants was associated to those with a history of hypertension at baseline. Specifically, over the first 12 months post-stroke, the proportion of participants whose BP readings were hypertensive doubled (27–51%) in those with a history of hypertension at baseline. In contrast, the proportion of participants whose BP readings were hypertensive remained low (0–8%) at follow-ups in those without a baseline history of hypertension. These observations were consistent with previous cohort studies reporting that stroke survivors with an pre-morbid diagnosis of hypertension had a higher risk of having uncontrolled BP over the first 12 months post-stroke (10, 12, 15). This increase in BP after discharge from inpatient rehabilitation may be due to several factors. Stroke survivors who are discharged home from inpatient rehabilitation often have some residual physical deficits and disabilities. Stroke survivors with residual deficits and disabilities are less likely to be physically active and more likely to be sedentary after stroke (36, 37). This overall reduction in physical activity levels and increased sedentary time may have contributed to increased BP after discharge from inpatient rehabilitation (38). Existing or newly diagnosed concurrent diseases, such as kidney failure, may also increase BP after stroke (39). Another potential factor is poor adherence to antihypertensive medication after discharge from inpatient rehabilitation, where BP monitoring and adherence to medications become primarily self-managed. Poor adherence to antihypertensive medications is common after stroke, with 45% of patients considered to be non-adherent within the first 6-month post-stroke (40). Acceptable adherence to antihypertensive medications is associated with increased likelihood of achieving BP targets (<140/90 mmHg) (41, 42), and associated with a 32% risk reduction of a post-stroke adverse cardiovascular event (i.e., recurrent stroke, myocardial infarction, unstable angina, and all-cause mortality) (40). Future studies should examine the potential effects of exercise, physical activity, and adherence to antihypertensive medications on BP after discharge from inpatient stroke rehabilitation settings.

One third of all DOSE participants, and half of those with a baseline history of hypertension, at 12-month post-stroke had BP readings that were considered hypertensive according to the 2020 Canadian Stroke Best Practice Recommendations (>140/90 mmHg, and > 130/80 mmHg if diabetic) (4). However, optimal BP targets for treatment remain unclear. Stroke survivors may benefit from further risk reduction of recurrent stroke with lower BP targets, such as <120 or < 130 mmHg SBP compared with <140 mmHg SBP (6, 7). The observed increase in mean SBP of 10 mmHg and DBP of 5 mmHg over the first 12 months after stroke is clinically relevant. Data from large prospective cohorts (*n* = 1,383,399) of non-stroke populations suggests that a 10 mmHg reduction in SBP or 5 mmHg reduction in DBP were associated with a 30–40% risk reduction of stroke (43). Given that BP of all DOSE participants continued to increase after stroke, further investigation on optimal BP targets after inpatient rehabilitation is needed (4, 9, 44).

A key strength to the current study is that we included stroke survivors from six distinct rehabilitation hospitals and three provinces across Canada, with varying practices in rehabilitation and BP control by rehabilitation or community physicians in each setting. This improves the generalizability of our study findings. However, this study has some limitations. BP was only measured once as part of the safety screening for assessments at each timepoint. Multiple measures (at least 2) are recommended by most national hypertension guidelines for clinical research and diagnosis of hypertension (45, 46). Furthermore, ambulatory, 24-h monitoring of BP may be more accurate in determining changes in BP compared with the automated devices used in the DOSE trial (11, 47). BP was measured after completing the MoCA, which may have influenced stress levels and subsequent BP readings. The current study was a secondary analysis from the DOSE trial and was not powered to examine changes in BP with a relatively small sample size. This precluded further analyses and descriptions of different BP trajectories based on known factors, such as age groups (48), and observing any potential interaction effects between time and history of hypertension for SBP and presence of hypertension. While we included stroke type into the analyses, the small sample size may have also precluded observing any potential differences in BP trajectory between ischemic and hemorrhagic strokes due to difference in BP treatment guidelines (27). The exploratory nature of the current analysis and our small sample size may have also increased possibility for type-1 errors. Due to the eligibility criteria necessary for the DOSE study, the study sample may not be representative of the overall stroke rehabilitation population and limit generalizability of the results. While data on the types of medications was collected at baseline, we did not collect data on follow-up (i.e., 6- and 12-month post-stroke) antihypertensive medications and the adherence to antihypertensive medications. Therefore, the associated impact of antihypertensive medication adherence on BP changes could not be included in the current analysis.

## 5. Conclusion

For DOSE participants, BP increased over the first 12 months post-stroke. This trajectory was not influenced by whether or not participants received the 4-week DOSE interventions. Participants with a baseline history of hypertension were more likely to have increased BP and be hypertensive over the first 12 months post-stroke. Further investigation on optimal BP control strategies for stroke survivors discharged from inpatient rehabilitation is needed, including the role of high repetition gait training at aerobic intensities.

## Data availability statement

The original contributions presented in the study are included in the article/Supplementary material, further inquiries can be directed to the corresponding author.

## Ethics statement

The studies involving humans were approved by the University of British Columbia Clinical Research Ethics Board. The studies were conducted in accordance with the local legislation and institutional requirements. The participants provided their written informed consent to participate in this study.

## Author contributions

SH led the data analysis and preparation of the manuscript. CT assisted with manuscript preparation, and reviewed and approved the manuscript. TK led and designed the study, collected the data, and reviewed and approved the manuscript. AS advised and contributed to data analysis. MB, SD, MH, AK, SP, MP, and JY contributed to study design, data collection, and reviewed and approved the manuscript. JE supervised the study conception and design, data collection, manuscript preparation, and reviewed and approved of the manuscript. All authors contributed to the article and approved the submitted version.

## Funding

This work was supported by the Canadian Institutes of Health Research (Doctoral award to TK; Operating Grant FDN 143340 to JE); Canada Research Chair Program (JE); Heart and Stroke Foundation Canadian Partnership for Stroke Recovery Operating Grant (JE); and Michael Smith Health Research BC (Trainee award to SH). The funders of the study had no role in study design, data collection, data analysis, data interpretation, or writing of the report.

## Conflict of interest

The authors declare that the research was conducted in the absence of any commercial or financial relationships that could be construed as a potential conflict of interest.

## Publisher’s note

All claims expressed in this article are solely those of the authors and do not necessarily represent those of their affiliated organizations, or those of the publisher, the editors and the reviewers. Any product that may be evaluated in this article, or claim that may be made by its manufacturer, is not guaranteed or endorsed by the publisher.
