# Supplementary material for: Blood pressure trajectory of inpatient stroke rehabilitation patients from the Determining Optimal Post-Stroke Exercise (DOSE) trial over the first 12 months post-stroke
Source: Front Neurol. 2023 Sep 19;14:1245881. doi: 10.3389/fneur.2023.1245881 (PMC10546336; doi:10.3389/fneur.2023.1245881)
Supplement: Supplementary file 1 [file Table_1.docx]

Supplementary Material

**Supplemental Table 1: Sensitivity analysis with influential observations removed for the effect of intervention group on blood pressure over the first 12 months post-stroke**

| Predictors | Systolic Blood Pressure | | | Diastolic Blood Pressure | | | Hypertension | | |
| --- | --- | --- | --- | --- | --- | --- | --- | --- | --- |
|  | Est. | 95% CI | p | Est. | 95% CI | p | Odds Ratio | 95% CI | p |
| Intercept | 121.62 | 117.04 – 126.19 | **<0.001** | 73.66 | 70.43 – 76.90 | **<0.001** | 0.08 | 0.02 – 0.36 | **0.001** |
| Group (DOSE1)* | 0.32 | -5.55 – 6.19 | 0.914 | -0.39 | -4.55 – 3.76 | 0.852 | 1.46 | 0.26 – 8.23 | 0.670 |
| Group (DOSE2)* | 0.69 | -5.19 – 6.56 | 0.818 | 0.65 | -3.50 – 4.80 | 0.759 | 0.89 | 0.15 – 5.15 | 0.900 |
| Post-Intervention^¥^ | 4.21 | 1.18 – 7.23 | **0.007** | 1.28 | -0.72 – 3.28 | 0.209 | 1.51 | 0.52 – 4.40 | 0.451 |
| 6-Months Stroke^¥^ | 6.26 | 3.13 – 9.39 | **<0.001** | 3.93 | 1.88 – 5.97 | **<0.001** | 3.39 | 1.16 – 9.92 | **0.026** |
| 12-Months Post-Stroke^¥^ | 9.26 | 5.96 – 12.56 | **<0.001** | 4.87 | 2.73 – 7.01 | **<0.001** | 3.52 | 1.12 – 11.07 | **0.031** |
| **Random Effects** | | | | | | | | | |
| σ^2^ | 79.21 | | | 33.71 | | | 3.29 | | |
| τ_00_ _ID_ | 83.80 | | | 43.65 | | | 5.88 | | |
| ICC | 0.51 | | | 0.56 | | | 0.64 | | |
| N _ID_ | 74 | | | 74 | | | 74 | | |
| Observations | 253 | | | 254 | | | 260 | | |
| Marginal R^2^ / Conditional R^2^ | 0.063 / 0.545 | | | 0.049 / 0.586 | | | 0.034 / 0.653 | | |

*: Reference is the Usual Care group; ¥: Reference is Baseline timepoint. Bold values indicate statistical significance at p <0.05.

**Supplemental Table 2: Sensitivity analysis with influential observations removed for the longitudinal analyses of blood pressure from the linear mixed effects model over the first year post-stroke**

| Predictors | Systolic Blood Pressure | | | Diastolic Blood Pressure | | | Hypertension | | |
| --- | --- | --- | --- | --- | --- | --- | --- | --- | --- |
|  | Est. | 95% CI | p | Est. | 95% CI | p | Odds Ratio | 95% CI | p |
| (Intercept) | 115.54 | 111.89 – 119.20 | **<0.001** | 72.66 | 69.53 – 75.80 | **<0.001** | 0.01 | 0.00 – 0.05 | **<0.001** |
| Time in weeks since stroke | 0.17 | 0.11 – 0.23 | **<0.001** | 0.09 | 0.05 – 0.13 | **<0.001** | 1.03 | 1.01 – 1.05 | **0.004** |
| History of hypertension* | 12.48 | 8.09 – 16.86 | **<0.001** | 4.75 | 1.17 – 8.33 | **0.009** | 42.38 | 6.19 – 290.31 | **<0.001** |
| Age in years | 0.04 | -0.14 – 0.22 | 0.666 | -0.20 | -0.35 – -0.05 | **0.008** | 1.00 | 0.94 – 1.07 | 0.947 |
| Hemorrhagic Stroke¥ | -3.64 | -8.88 – 1.60 | 0.173 | -1.50 | -5.78 – 2.77 | 0.489 | 0.27 | 0.05 – 1.40 | 0.119 |
| **Random Effects** | | | | | | | | | |
| σ^2^ | 80.45 | | | 34.93 | | | 3.29 | | |
| τ_00_ _ID_ | 47.00 | | | 36.67 | | | 3.34 | | |
| ICC | 0.37 | | | 0.51 | | | 0.50 | | |
| N _ID_ | 74 | | | 74 | | | 74 | | |
| Observations | 253 | | | 255 | | | 263 | | |
| Marginal R^2^ / Conditional R^2^ | 0.260 / 0.533 | | | 0.125 / 0.573 | | | 0.343 / 0.674 | | |

*: Reference is no history of hypertension; ¥: Reference is ischemic stroke. Bold values indicate statistical significance at p <0.05.

**Supplemental Table 3 – Predictors of participants who missed 6-month and 12-month assessments**

| Predictors | **Participants who missed 6-month and 12-month assessments** | | |
| --- | --- | --- | --- |
|  | **Odds Ratios** | 95% CI | p |
| (Intercept) | 0.00 | 0.00 – 0.20 | **0.015** |
| Age | 1.04 | 0.98 – 1.11 | 0.176 |
| Female Sex | 2.00 | 0.56 – 7.31 | 0.282 |
| Baseline SBP | 1.04 | 1.00 – 1.09 | 0.059 |
| Observations | 72 | | |
| R^2^ Tjur | 0.191 | | |

SBP: systolic blood pressure. Bold values indicate statistical significance at p <0.05.
